# Circulating Testosterone as the Hormonal Basis of Sex Differences in Athletic Performance

**DOI:** 10.1210/er.2018-00020

**Published:** 2018-07-13

**Authors:** David J Handelsman, Angelica L Hirschberg, Stephane Bermon

**Affiliations:** 1 ANZAC Research Institute, University of Sydney, Concord, New South Wales, Australia; 2 Department of Andrology, Concord Hospital, Sydney, New South Wales, Australia; 3 Department of Women’s and Children’s Health, Karolinska Institutet, Stockholm, Sweden; 4 Department of Gynecology and Reproductive Medicine, Karolinska University Hospital, Stockholm, Sweden; 5 Laboratoire Motricité Humaine, Education, Sport, Santé, Université Côte d’Azur, Nice, France; 6 Health and Science Department, International Association of Athletics Federations, Monaco

## Abstract

Elite athletic competitions have separate male and female events due to men’s physical advantages in strength, speed, and endurance so that a protected female category with objective entry criteria is required. Prior to puberty, there is no sex difference in circulating testosterone concentrations or athletic performance, but from puberty onward a clear sex difference in athletic performance emerges as circulating testosterone concentrations rise in men because testes produce 30 times more testosterone than before puberty with circulating testosterone exceeding 15-fold that of women at any age. There is a wide sex difference in circulating testosterone concentrations and a reproducible dose-response relationship between circulating testosterone and muscle mass and strength as well as circulating hemoglobin in both men and women. These dichotomies largely account for the sex differences in muscle mass and strength and circulating hemoglobin levels that result in at least an 8% to 12% ergogenic advantage in men. Suppression of elevated circulating testosterone of hyperandrogenic athletes results in negative effects on performance, which are reversed when suppression ceases. Based on the nonoverlapping, bimodal distribution of circulating testosterone concentration (measured by liquid chromatography–mass spectrometry)—and making an allowance for women with mild hyperandrogenism, notably women with polycystic ovary syndrome (who are overrepresented in elite athletics)—the appropriate eligibility criterion for female athletic events should be a circulating testosterone of <5.0 nmol/L. This would include all women other than those with untreated hyperandrogenic disorders of sexual development and noncompliant male-to-female transgender as well as testosterone-treated female-to-male transgender or androgen dopers.

**Essential Points**

- It is widely accepted that elite athletic competitions should have separate male and female events
- The main justification is that men’s physical advantages in strength, speed, and endurance mean that a protected female category, with objective entry criteria, is required
- Prior to puberty, there is no sex difference in circulating testosterone concentrations and athletic performance
- From male puberty onward, the sex difference in athletic performance emerges as circulating testosterone concentrations rise as the testes produce 30 times more testosterone than before puberty, resulting in men having 15- to 20-fold greater circulating testosterone than children or women at any age
- This wide, bimodal sex difference in circulating testosterone concentrations and the clear dose-response relationships between circulating testosterone and muscle mass and strength, as well as the hemoglobin level, largely account for the sex differences in athletic performance
- Based on the nonoverlapping, bimodal distribution of circulating testosterone concentration (measured by liquid chromatography–mass spectrometry) with 95% references ranges of 7.7 to 29.4 nmol/L in healthy men and 0 to 1.7 nmol/L in healthy premenopausal women—making an allowance for women with the mild hyperandrogenism of polycystic ovary syndrome, who are overrepresented in elite athletics—the eligibility criterion for female athletic events should be a circulating testosterone concentration of <5.0 nmol/L

Virtually all elite sports are segregated into male and female competitions. The main justification is to allow women a chance to win, as women have major disadvantages against men who are, on average, taller, stronger, and faster and have greater endurance due to their larger, stronger muscles and bones as well as a higher circulating hemoglobin level. Hence, elite female competition forms a protected category with entry that must be restricted by an objective eligibility criterion related, by necessity, to the relevant sex-specific physical advantages. The practical need to establish an eligibility criterion for elite female athletic competition led the International Association of Athletic Federations (IAAF) to establish a rule in 2011, endorsed by the International Olympic Committee (IOC) in 2012, for hyperandrogenic women. That IAAF regulation stated that for athletes to be eligible to compete in female events, the athlete must be legally recognized as a female and, unless she has complete androgen insensitivity, maintain serum testosterone <10 nmol/L. That IAAF eligibility rule was challenged by an athlete to the Court for Arbitration in Sports, which ruled in 2015 that, although an eligibility criterion was justified, there was insufficient evidence of the extent of the competitive advantage enjoyed by hyperandrogenic athletes who had circulating testosterone >10 nmol/L over female athletes with circulating testosterone in the normal female range. The Court for Arbitration in Sports suspended the rule pending receipt of such evidence. In that context, the present review presents the available evidence on the hormonal basis for the sex difference in athletic performance. It concludes that the evidence justifies a revised eligibility criterion of a threshold circulating testosterone concentration of 5 nmol/L (measured by a mass spectrometry method).

## Sex, Fairness, and Segregation in Sport

If sports are defined as the organized playing of competitive games according to rules (1), fixed rules are fundamental in representing the boundaries of fair sporting competition. Rule breaking, whether by breaching eligibility or competition rules, such as use of banned drugs, illegal equipment, or match fixing, creates unfair competitive advantages that violate fair play. Cheating constitutes a fraud against not just competitors but also spectators, sponsors, the sport, and the public. In the absence of genuine fair competition, elite sports would lose their wide popular appeal and ability to captivate and inspire with the authentic attraction of genuine contest between highly trained athletes.

Nevertheless, fairness is an elusive, subjective concept with malleable boundaries that may change over time as social concepts of fairness evolve. For example, until the late 19th century when organized sports trainers emerged, training itself was considered a breach of fairness because competition was envisaged at that time as a contest based solely on natural endowments. Similarly, sports once distinguished between amateurs and professionals. The concept of fairness has deep and complex philosophical roots mainly focused on notions of distributive justice. These considerations affect sports through the universal application of antidiscrimination and human rights legislation. Less attention is given to the philosophical basis of fair competition in elite sports, where the objectives are not egalitarian but aim to discover a hierarchy of achievement derived from a mixture of unequal natural talent and individual training effort. Excellent, insightful discussion of the legal and moral complexities of sex and fair competition in elite sports from a legal scholar and former elite female athlete is available (2).

The terms *sex* and *gender* are often confused and used as if interchangeable. *Sex* is an objective, specific biological state, a term with distinct, fixed facets, notably genetic, chromosomal, gonadal, hormonal, and phenotypic (including genital) sex, each of which has a characteristic defined binary form. Whereas all facets of biological sex are almost always aligned so that assignment of sex at birth is straightforward, rare instances in which two or more facets of biological sex conflict constitute an intersex state, now referred to as disorders (or differences) of sex development (DSDs) (3). In contrast, *gender* is a subjective, malleable, self-identified social construct that defines a person’s individual gender role and orientation. Prompted by biological, personal, and societal factors, volitional expression of gender can take on virtually any form limited only by the imagination, with some individuals asserting they have not just a single natal gender but two genders, none, a distinct third gender, or gender that varies (fluidly) from time to time. Hence, whereas gender is usually consistent with biological sex as assigned at birth, in a few it can differ during life. For example, if gender were the basis for eligibility for female sports, an athlete could conceivably be eligible to compete at the same Olympics in both female and male events. These features render the unassailable personal assertion of gender identity incapable of forming a fair, consistent sex classification in elite sports.

The strongest justification for sex classification in elite sports is that after puberty men produce 20 times more testosterone than women (4–7), resulting in circulating testosterone concentrations 15-fold higher than in children or women of any age. Age-grade competitive sporting records show no sex differences prior to puberty, whereas from the age of male puberty onward there is a strong and ongoing male advantage (8). The striking male postpubertal increase in circulating testosterone provides a major, ongoing, cumulative, and durable physical advantage in sporting contests by creating larger and stronger bones, greater muscle mass and strength, and higher circulating hemoglobin as well as possible psychological (behavioral) differences. In concert, these render women, on average, unable to compete effectively against men in power-based or endurance-based sports.

Sex classification in sports therefore requires proof of eligibility to compete in the protected (female) category. This deceptively simple requirement for fairness is taken for granted by peer female competitors who regard participation by males, or athletes with physical features closely resembling males, as unfair. This makes policing of eligibility inescapable for sports, to avoid unfair male participation in female events. However, such policing inevitably intrudes into highly personal matters so that it must be achieved with respect for dignity and privacy, demanding use of the least invasive, scientifically reliable means. Unsurprisingly, this dilemma has always been highly contentious since it first entered international elite sports in the early 20th century, and it has become increasingly prominent and contentious in recent decades; nevertheless, the requirement to maintain fair play in female events will not disappear as long as separate female competitions exist. During recent decades, there has been progressively better understanding of the complex biology of genetic sex determination and the impact of pubertal sexual maturation in establishing phenotypic sexual dichotomy in physical capabilities. These sex-dichotomous physical features form the basis of, but remain quite distinct from, adult gender roles and identity. During the last century, as knowledge grew, the attempts to formalize a scientific basis for the unavoidable necessity of policing eligibility for the female category have been continually challenged. Most recently, the increasing assertion of gender self-identification as a social criterion has further challenged the hegemony of biology for determining “sports sex,” Coleman’s apt term (2). Allowing subjective gender self-identification to become the sole criterion of sports sex would allow for gaming and perceptions of systematic unfairness to grow. The case for women’s sports being defined by sex rather than gender, including the consequences of acceding to gender-based classification, has been outlined (9) in arguing the importance of proper medical management of athletes intending to compete in female events.

Separate male and female events in sports is a dominant form of classification that is superimposed on other graduated age group and weight classifications (*e.g.*, in weightlifting, power lifting, wrestling, boxing, rowing), which reflect differences in strength, power, and speed to ensure fairness in terms of opportunity to win and, additionally, safety in contact sports. Age and weight classifications rely on objective criteria (birth date, weigh-in weight) for eligibility, and so should sex classification. Nevertheless, some power sports dependent on explosive strength and power (*e.g.*, throwing events, sprinting) do not segregate weight classes, whereas other sports where height is an advantage (*e.g.*, basketball, jockeys) do not have height classifications. These sports disproportionately attract athletes with greater weight and/or power-to-weight ratio or advantageous stature, respectively. If sex classification were eliminated, such open or mixed competitions would be dominated almost exclusively by men. It therefore seems highly unlikely that sex classification would ever be discarded, despite calls on philosophical or sociological grounds to end “gender” classification in sport (10).

## Sex Difference in Circulating Testosterone Levels

### Testosterone biosynthesis, secretion, and regulation in men and women

An androgen is a hormone capable of developing and maintaining masculine characteristics in reproductive tissues (notably the genital tract, as well as in other tissues and organs associated with secondary sexual characteristics and fertility) and contributing to the anabolic status of nonreproductive body tissues (11). The two dominant bioactive androgens circulating in mature mammals, including humans—testosterone and its more potent metabolite DHT—account for the development and maintenance of all androgen-dependent characteristics, and their circulating levels in men and nonpregnant women arise from steroids synthesized *de novo* in the testes, ovary, or adrenals (12).

The sexually undifferentiated gonads in the embryo develop into either ovaries or testes according to whether a Y chromosome (or at least the *sry* gene) is present. After birth and until puberty commences, circulating testosterone concentrations are essentially the same in boys and girls, other than briefly in the neonatal period of boys when higher levels prevail. The onset of male puberty, a brain-driven process triggered by a still mysterious hypothalamic or higher cerebral mechanism (13), initiates a hormonal cascade. In males, this leads to enhanced pituitary LH secretion that stimulates the 500 million Leydig cells in the testes to secrete 3 to 10 mg (mean, 7 mg) of testosterone daily (4, 6, 7, 14, 15). This creates a very high local concentration of testosterone within the testis as well as a steep downhill concentration gradient into the bloodstream that maintains circulating testosterone levels at adult male levels, which are tightly regulated by strong negative hypothalamic feedback of circulating testosterone. In the absence of testes, these mechanisms do not function in females. In girls, serum testosterone increases during puberty (16), peaking at age 20 to 25 years before declining gradually with age (17, 18), but it remains <2 nmol/L at all ages, as determined by a reliable method (see below).

In adult women, circulating testosterone is derived from three roughly equal sources: direct secretion from the adrenal gland or the ovary and indirect extraglandular conversion (in liver, kidney, muscle, fat, skin) from testosterone precursors secreted by the adrenal and ovary. Only when circulating testosterone concentrations rise in male adolescents above the prepubertal concentrations does the virilization characteristic of men commence, progress, and endure throughout adult life, at least until old age (18). In combination, these different sources produce ∼0.25 mg of testosterone daily so that throughout life women maintain circulating testosterone levels of <2 nmol/L. Circulating testosterone concentrations in women are subject to little dynamic physiological regulation. As a result, circulating testosterone concentrations in healthy premenopausal women are stable (nonfluctuating) and not subject to strong negative feedback by exogenous testosterone (as happens in men). Even the small rise (50%) at the time of the mid-cycle LH surge triggering ovulation (19) remains within the physiological range for premenopausal females.

### Male and female reference ranges for circulating testosterone

A reliable threshold for circulating testosterone must be set using measurement by the reference method of liquid chromatography–mass spectrometry (LC-MS) rather than using one of the various available commercial testosterone immunoassays. The necessary reliance on steroid mass spectrometry for clinical applications in endocrinology, reproductive medicine, and sports medicine is widely recognized. It has been standard for decades in antidoping science (20), and the growing consensus is that it is required for high-quality clinical research and practice recognized by cognate professional societies (21, 22) and editorials in leading clinical endocrinology (23) and reproductive medicine (24) journals. The inherently limited specificity of testosterone immunoassays arises from antibody cross-reactivity with structurally related steroids (such as precursors and metabolites) other than the intended target. As a result, all steroid immunoassays, including for testosterone, display method-specific bias whereby, for example, the lower limit of a testosterone reference range in healthy young men varies from 7.3 to 12.6 nmol/L according to the immunoassay used, so that no consensus definition of a lower limit could be obtained independent of the commercial immunoassay method used (25). Furthermore, testosterone immunoassays are optimized for circulating levels in men but display increasing inaccuracy at the lower, by an order of magnitude, circulating testosterone concentrations in women or children. In contrast to immunoassays, LC-MS–based methods are highly specific and do not depend on proprietary antibodies. Using LC-MS–based measurements, method-specific bias can be avoided and a fixed consensus lower reference limit defined (Table 1). Hence, for the precision required in sports medicine, whether for eligibility criteria or antidoping applications, testosterone in serum must be measured by LC-MS methods.

**Table 1.** Serum Testosterone Measurements by LC-MS Methods in Studies of Healthy Men and Women

Study	Sample (Age 18–40 y)	N	Lower 95% CL (nmol/L)	Upper 95% CL (nmol/L)
Men				
Sikaris *et al.*, 2005 (25)	Elite, eugonadal	124	10.4	30.1
Turpeinen *et al.*, 2008 (26)	Convenience	30	10.1	31.2
Kushnir *et al.*, 2010 (27)	Convenience	132	7.2	24.2
Salameh *et al.*, 2010 (28)	Convenience	264	7.1	39.0
Neale *et al.*, 2013 (29)	Convenience	67	10.6	31.9
Kelsey *et al.*, 2014 (30)	Secondary pooled analysis	1058	7.2	25.3
Hart *et al.*, 2015 (31)	Birth cohort	423	7.4	28.0
Travison *et al.*, 2017 (32)	Pooled two cohorts	1656	7.9	31.1
Number-weighted mean			7.7	29.4
Women				
Turpeinen *et al.*, 2008 (26)	Convenience	32	0.8	2.8
Kushnir *et al.*, 2010 (27)	Convenience	104	0.3	2.0
Salameh *et al.*, 2010 (28)	Convenience	235	0.03	1.5
Haring *et al.*, 2012 (33)	Population-based	263	0.04	2.0
Neale *et al.*, 2013 (29)	Convenience	90	0	1.7
Bui *et al.*, 2013 (34)	Convenience	25	0.30	1.69
Rothman *et al.*, 2013 (19)	Convenience	31	0.4	0.92
Bermon and Garnier, 2017 (35)	Elite athletes	1652	0	1.62
Eklund *et al.*, 2017 (36)	Elite athletes and controls	223	0.26	1.73
Number-weighted mean			0.06	1.68

Abbreviation: CL, confidence limit.

Prior to puberty, levels of circulating testosterone as determined by LC-MS are the same in boys and girls (16). They remain lower than 2 nmol/L in women of all ages. However, from the onset of male puberty the testes secrete 20 times more testosterone resulting in circulating testosterone levels that are 15 times greater in healthy young men than in age-similar women. Using LC-MS measurement, circulating testosterone in adults has a strikingly nonoverlapping bimodal distribution with wide and complete separation between men and women. Table 1 (25–36) summarizes data from appropriate reported studies using mass spectrometry–based methods to measure serum testosterone in healthy men and women. Based on a number-weighted pooling with conventional 95% two-sided confidence limits of the eight available studies using LC-MS measurements of serum testosterone, the reference range for healthy young men (18 to 40 years) is 7.7 nmol/L to 29.4 nmol/L. Similarly, summarizing the nine available studies for healthy menstruating women under 40 years, the 95% (two-sided) reference range is 0 to 1.7 nmol/L. These reference limits do not control for factors such as oral contraceptive use (35, 36), menstrual phase (19), SHBG (37, 38), overweight (39, 40), fasting and smoking (41), diet (40), and physical activity (42, 43) in women and men, all of which have small effects on circulating testosterone but without materially influencing the divergence between the nonoverlapping bimodal distribution of male and female reference ranges of circulating testosterone.

In creating a threshold for eligibility for female events it is also necessary to make allowance for women with polycystic ovary syndrome (PCOS) and nonclassical adrenal hyperplasia. PCOS is a relatively common disorder among women of reproductive ages with a prevalence of 6% to 10%, depending on the diagnostic criteria used (44), in which mild hyperandrogenism is a key clinical feature and has higher than expected prevalence among elite female athletes (36, 45–47). Nonclassical adrenal hyperplasia is a milder and later (adult) onset variant of classical congenital adrenal hyperplasia (48) with a much higher but still rare population prevalence (1:1000 vs 1:16,000 for the classical variant) (49). Table 2 (50–64) summarizes clinical studies (n = 16, ≥40 women) reporting serum testosterone concentrations measured by LC-MS in samples from women with PCOS.

**Table 2.** Summary of Serum Testosterone (nmol/L) by LC-MS in Women With PCOS From 16 Studies

Study	N	Mean	SD
Moran *et al.*, 2017 (50)	92	*0.24*	*0.08*
Münzker *et al.*, 2017 (51)	274	*0.93*	*0.19*
O'Reilly *et al.*, 2017 (52)	114	*0.55*	*0.19*
Handelsman *et al.*, 2017 (53)	152	0.38	0.25
Pasquali *et al.*, 2016 (54)	156	1.17	0.47
Yang *et al.*, 2016 (55)	1159	2.2	1.44
Tosi *et al.*, 2016 (56)	116	1.33	0.55
Daan *et al.*, 2015 (57)	170	*1.64*	*0.53*
Bui *et al.*, 2015 (58)	44	*0.85*	*0.3*
Keefe *et al.*, 2014 (59)	52	*1.7*	*0.97*
Yasmin *et al.*, 2013 (60)	165	1.99	1.02
Janse *et al.*, 2011 (61)	200	*1.12*	*0.47*
Jedel *et al.*, 2011 (62)	72	*0.23*	*0.08*
Legro *et al.*, 2010 (Mayo) (63)	596	*2.12*	*0.89*
Legro *et al.*, 2010 (Quest) (63)	596	*1.98*	*0.97*
Stener-Victorin *et al.*, 2010 (64)	74	1.53	0.62
Sum	4032		
Number-weighted mean		1.69	0.87

Data taken directly from paper or interpolated from other data (*e.g.*, median, quartiles, ranges, sample size) supplied as described by Wan *et al.,* 2014 (Estimating the sample mean and standard deviation from the sample size, median, range and/or interquartile range. BMC Med Res Methodol 14:135) are shown in italics.

The pooled data reveal that the upper limit of serum testosterone in women with PCOS is 3.1 nmol/L (95% CI, one-sided) or 4.8 nmol/L (using a 99.99% CI, one-sided) (Table 3). Hence, a conservative threshold for circulating testosterone of 5 nmol/L measured by LC-MS would identify <1:10,000 women with PCOS as false positives, based on circulating testosterone measurement alone. Circulating testosterone higher than this threshold is likely to be due to testosterone-secreting adrenal or ovarian tumors, intersex/DSD, badly controlled or noncompliant male-to-female (M2F) transgender athletes, or testosterone doping.

**Table 3.** Upper Confidence Limits on Serum Testosterone in Women With PCOS

Confidence Interval	Likelihood*^a^*	SD*^b^*	One-Sided*^c^*	Two-Sided*^c^*
95%	1:20	1.96	3.13	3.39
99%	1:100	2.35	3.47	3.73
99.9%	1:1000	3.10	4.21	4.39
99.99%	1:10,000	3.72	4.77	4.95

^a^ Likelihood that a woman with PCOS would exceed that limit by chance.

^b^ Number of SDs for each confidence limit.

^c^ Two-sided CIs are conventional for a result that could exceed or fall below confidence limits, but here we focus only on values exceeding the upper limit, so that one-sided confidence limits are appropriate.

### The physiological effects of testosterone depend on the circulating testosterone, not its source (endogenous or exogenous)

Testosterone, whether of a natural endogenous or manufactured exogenous source, has an identical chemical structure and biological effects, aside from minor differences in isotopic composition, which are biologically insignificant. At equivalent doses and circulating levels, exogenous testosterone exerts the same biological and clinical effects on every known androgen-responsive tissue or organ as endogenous testosterone, apart from effects on spermatogenesis, which as discussed below is only a matter of degree. Consequently, exogenous testosterone is a fully effective substitute for endogenous testosterone in therapeutic use, countering the effects of testosterone deficiency due to hypogonadism (reproductive system disorders). Any purported differences between endogenous and exogenous testosterone are due to corresponding differences in the endogenous production rate or exogenous dose. Such differences in effective exposure lead to corresponding differences in circulating testosterone levels and its effects according to the dose-response curves for testosterone.

Similar to all hormones and drugs, over their effective range of biological activity the dose-response relationship for testosterone is usually a sigmoidal curve with lower and upper plateaus joined by a monotonically rising middle region, which may be linear in the natural scale but more often log-linear (linear on the log or similar transformed scale). In the middle portion of the typical sigmoidal dose-response curve for the same increase in testosterone dose (or concentration), the response would be increased in simple proportional (*i.e.*, linear) but more often on a logarithmic scale. In contrast, at the lower and upper plateaus of dose or concentrations, changes in testosterone exposure may evoke minimal or no response on the endpoint. For example, in women of any age circulating testosterone concentrations are along the lower plateau of the dose-response curve, so that increases in circulating testosterone concentrations within that lower plateau may have minimal or no effect. In female athletes with the mild hyperandrogenism of PCOS, higher performance has been shown (47), with their muscle mass and power performance correlating with androgen levels (36). However, beyond these effects where endogenous testosterone concentrations are in the high-normal adult female range, it is only when the increases in circulating testosterone concentrations substantially and consistently exceed those prevailing in childhood (<2 nmol/L) and among women including those with PCOS (<5 nmol/L) that the effects would replicate the effects of rising testosterone concentrations of boys in middle to late puberty (typically >8 nmol/L), that is, the masculinizing effects of increased muscle, bone, and hemoglobin characteristics of men. As shown above, the circulating testosterone of most women never reaches consistently >5 nmol/L, a level that boys must sustain for some time to exhibit the masculinizing effects of male puberty.

In addition, the effects of testosterone are modulated in a form of fine tuning by the patterns of exposure, such as whether the circulating testosterone is delivered in the unphysiological steady-state format (*e.g.*, quasi–steady-state delivery by implant or transdermal products) or by the peak-and-trough delivery of injections, as opposed to the natural state of endogenous fluctuations in serum testosterone around the average adult male levels. However, these latter pattern effects are subtle and the dominant effect remains that of dose and average testosterone concentrations in blood, however they arise. Furthermore, there is evidence that the androgen sensitivity of responsive tissues differs and may be optimal at different circulating testosterone concentrations (65).

Male sexual function is maintained by endogenous testosterone at adult male circulating concentrations. These effects can be replicated by exogenous testosterone if and only if it achieves comparable circulating testosterone concentrations. For example, in a well-controlled prospective study of older men with prostate cancer (66), androgen deprivation achieving castrate levels of circulating testosterone sustained during 12 months markedly suppressed sexual desire and function, whereas those effects did not occur in age-matched men having nonhormonal treatment of prostate cancer or those without prostate cancer. In healthy younger men whose endogenous testosterone was fully suppressed, sexual function completely recovered when circulating testosterone was restored to the physiological male range by administration of exogenous testosterone (67). Similar effects were also observed in healthy, middle-aged men in whom male sexual function was fully maintained (compared with placebo) during 2 years of treatment with an exogenous androgen (DHT) despite that treatment causing sustained, complete suppression of endogenous testosterone (68). This further supports the key interpretation that the biological effects of exogenous or endogenous testosterone are the same at comparable circulating levels.

Clinically, exogenous testosterone replicates fully all effects of endogenous testosterone on every reproductive and nonreproductive organ or tissue, with the sole exception of the testis. Sperm production in the testis requires a very high concentration of testosterone (typically 100-fold greater than in the general bloodstream), which is produced in nature only by the action of the pituitary hormone LH. LH stimulates the Leydig cells in the interstitial space of the testis between seminiferous tubules to produce high intratesticular concentrations of testosterone, which are necessary and sufficient to initiate and maintain sperm production in the adjacent seminiferous tubules. This high concentration of testosterone also provides a downhill gradient to supply the rest of the body, where circulating testosterone acts on androgen-responsive tissues to produce and maintain masculine patterns of androgenization. When exogenous testosterone (or any other androgen) is administered to men, pituitary LH is suppressed by negative feedback and the sperm production halts for as long as exogenous testosterone or androgen exposure continues, after which it recovers (69). However, even the reduction in spermatogenesis and testis size when men are treated with exogenous testosterone is only a matter of degree. It is well established in rodents (70, 71) that spermatogenesis is induced by exogenous testosterone when the testosterone concentrations in the testis are high enough to replicate what occurs naturally via LH stimulation (72). However, direct replication that high-dose testosterone also initiates and maintains spermatogenesis in humans is not feasible, as these testosterone doses are 10- to 100-fold higher than could be safely given to humans. Nevertheless, confirmatory evidence in humans is available from rare cases of men with an activating mutation of the chorionic gonadotropin/LH receptor (73, 74). This mutation causes autonomous testicular testosterone secretion leading to precocious puberty arising from the premature adult male circulating testosterone concentrations that lead to complete suppression of circulating gonadotropin (LH, FSH) secretion. In this illustrative case the testis was exposed to nonphysiologically high testosterone concentrations (but without any gonadotropin stimulation) that induced sperm production and allowed for natural paternity (73). This indicates that even for spermatogenesis, exogenous testosterone can replicate all biological effects of endogenous testosterone in accordance with the relevant dose-response characteristics.

The most realistic view is that increasing circulating testosterone from the childhood or female range to the adult male range will have the same physiological effects whether the source of the additional testosterone is endogenous or exogenous. This is strongly supported by well-established knowledge about the relationship of circulating testosterone concentrations with the timing and manifestations of male puberty. The characteristic clinical features of masculinization (*e.g.*, muscle growth, increased height, increased hemoglobin, body hair distribution, voice change) appear only if and when circulating testosterone concentrations rise into the range of males at mid-puberty, which are higher than in women at any age even after the rise in circulating testosterone in female puberty. If and only if the pubertal rise in circulating testosterone fails will the males affected be clinically considered hypogonadal. Such a failure of male puberty may occur for genetic reasons (arising from mutations that inactivate any of the cascade of proteins whose activity is critical in the hypothalamus to trigger male puberty) or as a result of acquired conditions, caused by pathological disorders of the hypothalamus or pituitary or functional defects arising from severe deficits of energy or nutrition (*e.g.*, extreme overtraining, undernutrition), with the latter being comparable with hypothalamic amenorrhea or anorexia nervosa in female athletes/ballet dancers. If male puberty fails, testosterone replacement therapy is fully effective in replicating all of the distinctive masculine features apart from spermatogenesis.

### Elevated circulating testosterone concentration caused by DSDs

Rare genetic intersex conditions known as DSDs can lead to markedly increased circulating testosterone in women. When coupled with ambiguous genitalia at birth, they may appear as undervirilized males or virilized females. This can cause athletes who were raised and identify as women to have circulating testosterone levels comparable to those of men and greatly exceeding those of non-DSD (and nondoped) women, including those with PCOS. Key congenital disorders in this category are 46,XY DSDs, namely 5*α* reductase deficiency (75), 17*β*-hydroxysteroid dehydrogenase type 3 deficiency (76), and androgen insensitivity (77, 78), as well as congenital adrenal hyperplasia (79), which is a 46,XX DSD. There is evidence that the first three conditions, components of 46,XY DSDs, are 140-fold more prevalent among elite female athletes than expected in the general population (80).

Genetic 5*α* reductase deficiency is due to an inactivating mutation in the 5*α* reductase type II enzyme (75). This leads to a deficit of DHT during fetal life when DHT is required for converting the sex-undifferentiated embryonic and fetal tissue to form the sex-differentiated masculine form external genitalia. Although genetic males (46,XY) with 5*α* reductase deficiency will develop testes, they usually remain undescended and labial fusion to form a scrotum and phallic growth does not occur. Hence, at birth the external genitalia may appear feminine, leading to a female assigned natal sex. Thus, individuals with 5*α* reductase deficiency may have male chromosomal sex (46,XY), gonadal sex (testes), and hormonal sex (adult male testosterone concentrations), but such severely undervirilized genitalia that affected individuals may be raised from birth as females rather than as undervirilized males. However, from the onset of male puberty, testicular Leydig cells start producing large amounts of testosterone, and the steep rise in circulating testosterone to adult male levels (with the permissive role of 5*α* reductase activity) leads to masculine virilization, including male patterns of muscle and bone growth, hemoglobin levels, and other masculine body habitus features (hair growth pattern, voice change), as well as phallic growth (80). Such changes of male puberty prompt around half affected individuals who had female sex assigned at birth and developed as girls prior to puberty to adopt a male gender identity and role at puberty (81). Sperm are formed in the testes so that, using *in vitro* fertilization, these individuals may father children (82).

17*β*-Hydroxysteroid dehydrogenase type 3 deficiency (76) has a natural history similar to that of 5*α* reductase deficiency. This disorder is due to inactivating mutations in a steroidogenic enzyme expressed only in the testis and that is essential for testosterone formation in the fetus. In the absence of a functional enzyme, the testis makes little testosterone but instead secretes large amounts of androstenedione, the steroid immediately prior to the enzymatic block. In the circulation, the excess of androstenedione is converted to testosterone (mainly by the enzyme AKR1C3) (12). Although the circulating testosterone is then converted to circulating DHT, insufficient DHT is formed locally within the urogenital sinus to virilize genitalia at birth. This causes the same severe undervirilization of the external genitalia of genetically male individuals, leading to ambiguous genitalia at birth despite male chromosomal, gonadal, and hormonal sex. When puberty arrives, the testes start producing the adult male testosterone output. Again, this leads to marked virilization and subsequent assumption of a male gender identity by some affected individuals, conflicting with a female assigned natal sex and childhood upbringing.

Androgen insensitivity, which arises from mutation in the androgen receptor (AR), poses different but complex challenges for eligibility for female athletic events. As the AR is located on the X chromosome, genetic males (46,XY) are hemizygous, so that an inactivating mutation in the AR can be partially or fully insensitive to androgen action. Affected individuals have male internal genitalia (testes in the inguinal canal or abdomen with Wolffian ducts) and consequently adult male circulating testosterone concentrations after puberty. These nonlethal mutations have a wide spectrum of functional effects, ranging from full resistance to all androgen action in complete androgen insensitivity syndrome (CAIS) where individuals have a full female phenotype with normal female external genitalia, to partial androgen insensitivity syndrome (PAIS) where some androgen action is still exerted, leading to various degrees of ambiguous genitalia, or to mild androgen insensitivity, which produces a very mild, undervirilized male phenotype (normal male genital and somatic development but with little body hair and no male pattern balding) (77). Testosterone (and dihydrotestosterone) have no consistent effect of inducing normal nitrogen retention (anabolic) responses in patients with CAIS (83–86), although some reduced androgen responsiveness is retained by patients with PAIS (84, 87–90). Athletes with CAIS can compete fairly as females because the circulating testosterone, although at adult male levels, has no physiological effect so that, in terms of androgen action and the ensuing physical somatic advantages of male sex, affected individuals are indistinguishable from females and gain no benefits of the sex difference arising from unimpeded testosterone action. A more complex issue arises with athletes having PAIS reflecting the degree of incomplete impairment of AR function. Residual androgen action in such AR mutations is harder to characterize quantitatively, as there is no standardized, objective *in vitro* test to quantify AR functionality. Hence, individuals with PAIS may have adult male circulating testosterone concentrations but variable androgen sensitivity. At present, determination of eligibility to compete in the female category requires a case-by-case evaluation, primarily based on the degree of virilization. The current best available clinical approach to determining the functional impact (degree of functionality/sensitivity) of an AR mutation is based on the degree of somatic, primarily genital, virilization assessed according to the Quigley classification of grade of androgen sensitivity (91).

Congenital adrenal hyperplasia (CAH) is a relatively common defect in adrenal steroidogenesis in the enzymatic pathway, leading to synthesis of cortisol, aldosterone, and sex steroid precursors. The disease varies in severity from life-threatening (adrenal failure) to mild (hirsutism and menstrual irregularity), or even asymptomatic and undiagnosed. The most common mutations causing CAH occur in the 21-hydroxylase enzyme, accounting for 95% of cases (79). The defect leads to a bottleneck, creating a major backing up of precursor steroids that then overflow into other steroid pathways, leading to diagnostic high levels of 17-hydroxyprogesterone and, in female patients, excessive circulating testosterone or other adrenal-source androgen precursors (*e.g.*, androstenedione, dehydroepiandrosterone) that may be converted to testosterone in tissues. A common clinical problem with management of CAH is that glucocorticoid/mineralocorticoid treatment is not always fully effective partly due to variable compliance, which may leave high circulating testosterone, including well into or even above the normal male range (92). It is unlikely that mild nonclassical congenital adrenal hyperplasia is a major contributor to the mild hyperandrogenism prevalent among elite female athletes. The prevalence of PCOS (6% to 16%) is about 100-fold higher than mild nonclassical congenital adrenal hyperplasia (0.1%) (49), whereas a disproportionately high number of elite female athletes (especially in power sports) have PCOS (45). In one study of hyperandrogenic female athletes, even mild nonclassic adrenal hyperplasia was ruled out by normal 17-hydroxyprogesterone (36) and, in another (47), reported serum androstenedione and cortisol did not differ from controls, ruling out significant congenital adrenal hyperplasia.

“Sex differences in height, where they exist, are largely dependent on postpubertal differences in circulating testosterone.”

## Sex Difference in Muscle, Hemoglobin, Bone, and Athletic Performance Relating to Adult Circulating Testosterone Concentrations

Following puberty, testosterone production increases (16) but remains <2 nmol/L in women, whereas in men testosterone production increases 20-fold (from 0.3 mg/d to 7 mg/d), leading to 15-fold higher circulating testosterone concentrations (15 vs 1 nmol/L). The greater magnitude of sex difference in testosterone production (20-fold) compared with circulating levels (15-fold) is due to women’s higher circulating SHBG, which retards testosterone clearance, creating a slower circulating half-time of testosterone. This order-of-magnitude difference in circulating testosterone concentrations is the key factor in the sex difference in athletic performance due to androgen effects principally on muscle, bone, and hemoglobin.

### Muscle

#### Biology

It has been known since ancient times that castration influences muscle function. Modern knowledge of the molecular and cellular basis for androgen effects on skeletal muscle involves effects due to androgen (testosterone, DHT) binding to the AR that then releases chaperone proteins, dimerizes, and translocates into the nucleus to bind to androgen response elements in the promoter DNA of androgen-sensitive genes. This leads to increases in (1) muscle fiber numbers and size, (2) muscle satellite cell numbers, (3) numbers of myonuclei, and (4) size of motor neurons (93). Additionally, there is experimental evidence that testosterone increases skeletal muscle myostatin expression (94), mitochondrial biogenesis (95), myoglobin expression (96), and IGF-1 content (97), which may augment energetic and power generation of skeletal muscular activity.

Customized genetic mouse models can provide unique experimental insight into mammalian physiology that is unobtainable by human experimentation. The tight evolutionary conservation of the mammalian reproductive system explains why genetic mouse models have provided consistent, high-fidelity replication of the human reproductive system (98, 99). Genetic males (46,XY) with androgen insensitivity displaying similar features occur through the spontaneous production of inactivating AR mutations in all mammalian species studied, including humans, where they are known as women with CAIS. The converse, genetic females (46,XX) resistant to all androgen action cannot occur naturally in humans or other mammals. This is because fully androgen-resistant females must have both X chromosomes carrying an inactivated AR. In turn, this requires acquiring one X chromosome from their father, and hemizygous males bearing a single X chromosome with an inactive AR produce no sperm, as a functional AR is biologically indispensable for making sperm in any mammal. However, androgen-resistant females can be bred by genetic engineering using the Cre-Lox system (100). An important finding from such studies is that androgen-resistant female mice have essentially the same muscle mass and function as wild-type androgen-sensitive females bearing normal AR, whereas androgen-resistant male mice have smaller and weaker muscle mass and function than do wild-type males and comparable instead with wild-type females (101). This indicates that androgen action, represented by circulating testosterone, is the key determinant of the higher muscle mass and strength characteristic of males compared with females. Furthermore, endogenous circulating testosterone has minimal effects on skeletal muscle mass and strength in female mice because of its low levels. Although these experiments cannot be replicated in humans, their key insight is that the higher circulating testosterone in males is the determinant of the male’s greater muscle mass and function compared with females. Nevertheless, there is also evidence that hyperandrogenic women, mostly with PCOS, have increased muscle mass and strength that correlates with mildly increased circulating testosterone in the high-normal female range (36, 47).

#### Observational data

There is a clear sex difference in both muscle mass and strength (102–104) even adjusting for sex differences in height and weight (104, 105). On average, women have 50% to 60% of men’s upper arm muscle cross-sectional area and 65% to 70% of men's thigh muscle cross-sectional area, and women have 50% to 60% of men’s upper limb strength and 60% to 80% of men’s leg strength (106). Young men have on average a skeletal muscle mass of >12 kg greater than age-matched women at any given body weight (104, 105). Whereas numerous genes and environmental factors (including genetics, physical activity, and diet) may contribute to muscle mass, the major cause of the sex difference in muscle mass and strength is the sex difference in circulating testosterone.

**Figure 1.**
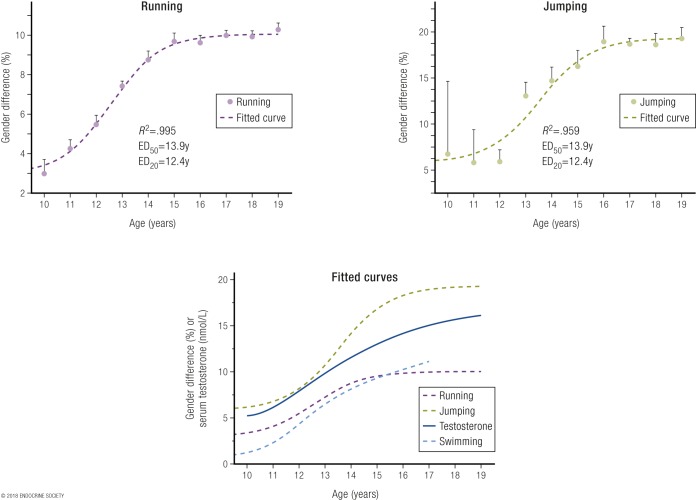
Sex differences in performance (in percentage) according to age (in years) in running events, including 50 m to 2 miles (upper left panel), and in jumping events, including high jump, pole vault, triple jump, long jump, and standing long jump (upper right panel) [for details, see Ref. (8)]. The lower panel is a fitted sigmoidal curve plot of sex differences in performance (in percentage) according to age (in years) in running, jumping, and swimming events, as well as the rising serum testosterone concentrations from a large dataset of serum testosterone of males. Note that in the same dataset, female serum testosterone concentrations did not change over those ages, remaining the same as in prepubertal boys and girls. Data are shown as mean and SEM of the pooled sex differences by age. Reproduced with permission from Handelsman DJ. Sex differences in athletic performance emerge coinciding with the onset of male puberty. *Clin Endocrinol (Oxf)*. 2017;**87**:68–72.

Age-grade competitive sports records show minimal or no female disadvantage prior to puberty, whereas from the age of male puberty onwards there is a strong and ongoing male advantage. Corresponding to the endogenous circulating testosterone increasing in males after puberty to 15 to 20 nmol/L (sharply diverging from the circulating levels that remain <2 nmol/L in females), male athletic performances go from being equal on average to those of age-matched females to 10% to 12% better in running and swimming events, and 20% better in jumping events (8) (Fig. 1). Corroborative findings are provided by a Norwegian study that examined performance of adolescents in certain athletic events but without reference to contemporaneous circulating testosterone concentrations (107). The striking postpubertal increase in male circulating testosterone provides a major, ongoing, cumulative, and durable advantage in sporting contests by creating greater muscle mass and strength. These sex differences render women unable to compete effectively against men, especially (but not only) in power sports.

These findings are supported by studies of nonathletic women showing that muscle mass is increased in proportion to circulating testosterone in women with mildly elevated testosterone levels due to PCOS (108, 109), a condition that is more prevalent among elite female athletes who exhibit these features (36, 45, 47), often undiagnosed (46), but that may provide an ergogenic advantage (47), consistent with the graded effects of circulating testosterone on explosive performance in men and women (110).

Studies of elite female athletes further corroborate these findings. One study demonstrates dose-response effects of better performance in some (400 m running, 400 m hurdles, 800 m running, hammer throw, pole vault) but not all athletic events correlated with significantly higher endogenous testosterone in female, but not male, athletes. Even within the low circulating testosterone levels prevailing within the normal female range, in these events there was a significant advantage of 1.8% to 4.5% among those in the highest tertile compared with the lowest tertile of endogenous testosterone (35). A further study of elite female athletes corroborates and extends these observations in that endogenous androgens are associated with a more anabolic body composition as well as enhanced muscular performance (36). In this study, 106 Swedish Olympic female athletes were compared with 117 age- and weight (body mass index)-matched sedentary control women for their muscle and bone mass (by dual-energy X-ray absorptiometry), their muscular strength (squat and countermovement jumps), and testosterone and DHT, as well as androgen precursors (dehydroepiandrosterone, androstenedione) and urinary androgen glucuronide metabolites (androsterone, etiocholanolone, 3 and 17 3*α*-diols) measured by LC-MS (36). The athletes displayed higher muscle (and bone) mass than did the sedentary control women, with strength tests correlating strongly with muscle mass whether in total or just in the legs. In turn, muscle mass and strength were correlated with androgens and androgen precursors. Considering that such studies may be confounded by factors such as menstrual phase and dysfunction, as well as heterogeneous sports disciplines, which weaken the power of the study, these findings can be regarded as quite robust.

#### Interventional data

Dose-response studies show that in men whose endogenous testosterone is fully suppressed, add-back administration of increasing doses of testosterone that produce graded increases in circulating testosterone causes a dose-dependent (whether expressed according to testosterone dose or circulating levels) increase in muscle mass (measured as lean body mass) and strength (65, 111). Taken together, these studies prove that testosterone doses leading to circulating concentrations from well below to well above the normal male range have unequivocal dose-dependent effects on muscle mass and strength. These data strongly and consistently suggest that the sex difference in lean body mass (muscle) is largely, if not exclusively, due to the differences in circulating testosterone between men and women. These findings have strong implications for power-dependent sport performance and largely explain the potent efficacy of androgen doping in sports.

**Figure 2.**
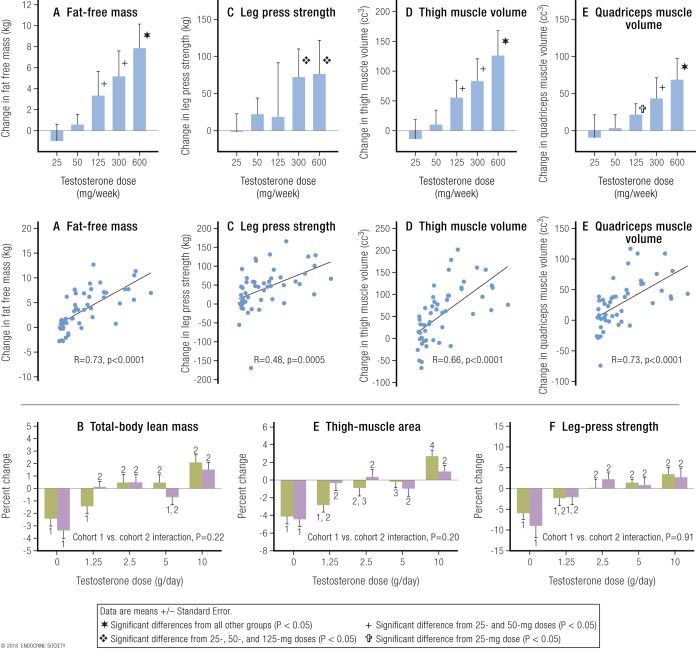
Strong dose-response relationship between testosterone dose and circulating concentration with muscle mass and strength in men. The upper panels [from Bhasin *et al.* (111)] display the strong dose-response relationships of muscle mass shown as (A) “lean” or “fat-free” mass or volume of (D) thigh and (E) quadriceps muscle and (C) of leg muscle strength with increasing testosterone dose (upper row) or circulating concentration (middle row). Serum testosterone concentrations are in US units (ng/dL; divide by 28.8 to get nmol/L). Adapted with permission from Bhasin S, Woodhouse L, Casaburi R, et al. Testosterone dose-response relationships in healthy young men. *Am J Physiol Endocrinol Metab*. 2001;**281**:E1172–E1181. The lower panels [from Finkelstein *et al.* (65)] show the strong dose-response relationships of (B) whole-body muscle mass, (E) thigh muscle mass, and (F) leg press strength with increasing testosterone dose. Cohorts 1 and 2 were treated with the same increasing doses of testosterone but either without (green fill, cohort 1) or with (purple fill, cohort 2) an aromatase inhibitor (anastrozole), which prevents conversion of testosterone to estradiol. The differences between cohorts (*i.e.*, use of anastrozole) was not significant for muscle mass and strength and can be ignored with results of the two cohorts being pooled. Reproduced with permission from Finkelstein JS, Lee H, Burnett-Bowie SA, Pallais JC, *et al.* Gonadal steroids and body composition, strength, and sexual function in men. N Engl J Med 2013;369:1011–1022.

The key findings providing conclusive evidence that testosterone has prominent dose-response effects in men are reported in studies by Bhasin and colleagues that proved a monotonic dose response, extending from subphysiological to supraphysiological range for men for testosterone effects on muscle mass, size, and strength in healthy young men, findings that have been replicated and confirmed by an independent group (65). Both sets of studies used a common design of fully suppressing all endogenous testosterone (to castrate levels) for the full duration of the experiment by administering a GnRH analog. In the Bhasin and colleagues studies, participants were then randomized to five groups and each received weekly injections of 25 mg, 50 mg, 125 mg, 300 mg, or 600 mg of testosterone enanthate for 20 weeks. In effect, this was two subphysiological and two supraphysiological testosterone doses. In these studies, the lowest testosterone dose produced a mean serum testosterone of 253 ng/dL (8.8 nmol/L) in younger men and 176 ng/dL (6.1 nmol/L) in older men. The studies showed a consistent dose response for muscle mass and strength that was clearly related to testosterone dose and consequential blood testosterone concentrations (Fig. 2, upper panel).

The study of Finkelstein *et al.* (65) involved the same design and involved 400 healthy men aged 20 to 50 years who had complete suppression of endogenous testosterone for the 16 weeks of the study, with testosterone added back using daily doses of 0, 1.25 g, 2.5 g, 5 g, or 10 g of a topical 1% testosterone gel. This again created a graded dose-response curve for serum testosterone and for muscle mass and strength. The inclusion of a 0 (placebo) dose allowed differentiation between the 0 and lowest testosterone dose. The placebo (0) dose produced a serum testosterone of 0.7 nmol/L (the typical mean for castrated men, childhood, and women of any age). Meanwhile, the lowest testosterone dose (1.25 g of gel per day) produced a serum testosterone of 6.9 nmol/L, which is equivalent to that of a male in early to middle puberty. A key finding for this review is that, from this study of men, the increase in serum testosterone from mean of normal female concentration (0.9 nmol/L) to supraphysiological female concentrations (6.9 nmol/L) produced significant increases of 2.3% for total body lean (muscle) mass, 3.0% for thigh muscle area, and 5.5% increase in leg press strength (digitized data pooling of both cohorts from lower panel, Fig. 2).

Studies of the ergogenic effects of supraphysiological concentrations of circulating testosterone require studies administering graded doses of exogenous testosterone for months. Owing to ethical concerns regarding risks of unwanted virilization and hormone-dependent cancers, however, few studies have administered supraphysiological testosterone doses to healthy women. One well-designed, randomized placebo-controlled study of postmenopausal women investigated the effects of different testosterone doses on muscle mass and performance and physical function (112). Sixty-two women (mean age, 53 years) all had a standard estrogen-replacement dose administered during a 12-week run-in period (to eliminate any hypothetical confounding effects of estrogen deficiency), after which they were randomized to one of five groups receiving weekly injections of testosterone enanthate (doses: 0, 3 mg, 6.25 mg, 12.5 mg, and 25 mg, respectively) for 24 weeks. The increasing doses of testosterone produced an expected dose response in serum testosterone concentrations (by LC-MS), with the highest testosterone dose (25 mg/wk) producing a mean nadir concentration of 7.3 nmol/L. The women whose testosterone concentrations were increased to 7.3 nmol/L achieved significant increases in muscle mass and strength (Table 4), ranging from 4.4% for muscle (lean) mass to between 12% and 26% for measures of muscle strength (chest and leg press, loaded stair climb). As muscle strength measurement is effort-dependent, the placebo-controlled design of the Huang *et al.* (112) study supports the further interpretation that the highest dose of testosterone also had prominent mental motivational effects in the effort-dependent tests of muscle strength. These findings provide salient direct evidence of the ergogenic effects of hyperandrogenism in female athletes confirming that at least up to average circulating testosterone concentrations of 7.3 nmol/L, women display a dose-response relationship similar to that of men, with supraphysiological doses of testosterone leading to significant gains in muscle mass and power.

**Table 4.** Effects of Testosterone on Muscle Mass and Strength in Women

Androgen-Sensitive Variable	Baseline	Increase	% Increase
Lean muscle mass, kg	43 ± 6	1.9 ± 0.5	4.4
Chest press, W	100 ± 26	26 ± 7	26
Leg press, N	744 ± 172	90 ± 30	12
Loaded stair-climb power, W	406 ± 77	56 ± 13	14

With data from Huang G, Basaria S, Travison TG, *et al.* Testosterone dose-response relationships in hysterectomized women with or without oophorectomy: effects on sexual function, body composition, muscle performance and physical function in a randomized trial. Menopause 2014;21:612–623. Data are shown as mean and SEM derived from Table 1 and digitized from Figure 4 from Huang *et al.* (112) showing the effects of testosterone (mean circulating concentration, 7.3 nmol/L) on muscle mass and strength in women treated with the highest testosterone dose (n = 11; 25 mg of testosterone enanthate per week).

**Figure 3.**
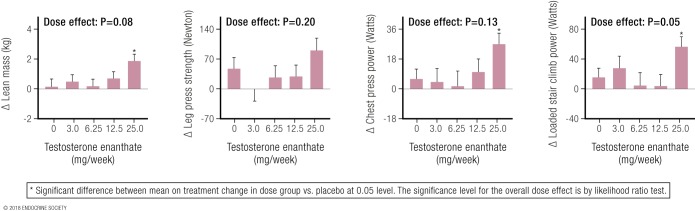
From Huang *et al.* (112): Dose-response effects on lean (muscle) mass and three measures of muscle strength as a result of increasing doses of weekly testosterone enanthate injections in women. Note the effects on all four parameters (three statistically significant) of the highest testosterone dose, the only one that produced circulating testosterone levels exceeding the normal female range. Reproduced with permission from Huang G, Basaria S, Travison TG, *et al.* Testosterone dose-response relationships in hysterectomized women with or without oophorectomy: effects on sexual function, body composition, muscle performance and physical function in a randomized trial. Menopause 2014;21:612–623.

These effects of testosterone administration on circulating testosterone concentrations and muscle mass and strength in females may be compared with the effects in males from the Finkelstein *et al.* (65) and Bhasin and colleagues studies. In men, the lowest testosterone dose (1.25 g/d) increased mean serum testosterone to 6.9 nmol/L (equivalent to levels seen in early to middle male puberty), resulting in significant increases of total body lean (muscle) mass (2.3%), thigh muscle area (3.0%), and leg press strength (5.5%) compared with the placebo dose that resulted in a serum testosterone of 0.7 nmol/L. In the Huang *et al.* (112) study (Fig. 3), muscle mass and strength in postmenopausal women displayed a flat response at the three lower doses, when circulating testosterone concentrations remain <5 nmol/L, and displayed a significant increase only when the mean circulating testosterone concentration produced by the highest testosterone dose first increased circulating testosterone concentrations >5 nmol/L. This pattern, flat at lower doses and rising at the highest dose, represents the lower plateau and the earliest rising portion, respectively, of the sigmoidal dose-response curve of testosterone for muscle.

Data corroborating the Huang *et al.* study results comes from another well-controlled study in which postmenopausal women who were administered methyl testosterone following a run-in period of estrogen replacement displayed a significant increase in lean (muscle) mass as well as upper and lower limb power during a 16-week double-blind, parallel group study (113).

Similarly, two prospective studies of the first 12 months of treatment of transmen [female-to-male (F2M) transgender] shows a consistent major increase in muscle mass and strength due to testosterone administration. In one study testosterone treatment of 17 transmen achieving adult male circulating testosterone levels (mean, 31 nmol/L) increased muscle mass by 19.2% (114). In a second study, 23 transmen administered adult male testosterone doses also produced striking increases in total body muscle size and limb muscle size (by 6.5% to 16.6%) and grip strength (by 18%) compared with age-matched untreated control women (115). Conversely, testosterone suppression (using an estrogen-based treatment regimen) in 20 transwomen (M2F transgender) that reduced circulating testosterone levels from adult male range to adult female range led to a 9.4% reduction in muscle mass (measured as cross-sectional area).

#### Effects on athletic performance

Muscle growth, as well as the increase in strength and power it brings, has an obvious performance-enhancing effect, in particular in sports that depend on strength and (explosive) power, such as track and field events (107, 110). There is convincing evidence that the sex differences in muscle mass and strength are sufficient to account for the increased strength and aerobic performance of men compared with women and is in keeping with the differences in world records between the sexes (116). The basis for the sex difference in muscle mass and strength is the sex difference in circulating testosterone as clearly shown (for example) by (1) the enhanced athletic performance of men compared with prepubertal boys and women (8); (2) the close correspondence of muscle growth (muscle size) with muscle strength in ascending dose studies in men by Bhasin *et al.* (111, 117–119) and Finkelstein *et al.* (65) and in postmenopausal women by Huang *et al.* (112); (3) the effect of male castration in reducing muscle size and strength, effects that are fully rectified by testosterone replacement; and (4) the striking efficacy of androgen doping on the sports performances of German Democratic Republic female athletes (120).

### Hemoglobin

#### Biology

It is well known that levels of circulating hemoglobin are androgen-dependent and consequently higher in men than in women by 12% on average; however, the physiological mechanism by which androgens such as testosterone boosts circulating hemoglobin is not fully understood (121). Testosterone increases secretion of and sensitivity to erythropoietin, the main trophic hormone for erythrocyte production and thereby hemoglobin synthesis, as well as suppressing hepcidin (122), a crucial iron regulatory protein that governs the body’s iron economy. Hepcidin has to balance the need for iron absorption from foods (the only source of iron required for the body’s iron-containing proteins) against the fact that the body has no mechanism to shed excess iron, which can be toxic. Adequate iron availability is essential for normal erythropoiesis and synthesis of key heme, iron-containing oxygen-transporting proteins such as hemoglobin and myoglobin (123) as well as other iron-dependent proteins such as cytochromes and DNA synthesis and repair enzymes. Experimental evidence in mice shows that testosterone increases myoglobin content of muscle with potential for augmenting aerobic exercise performance (96), but this has not been evaluated in humans.

**Figure 4.**
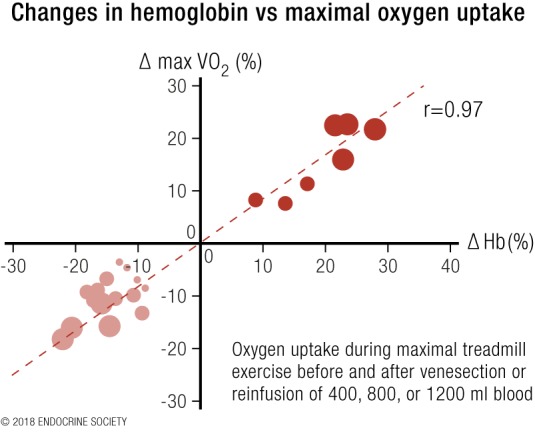
Redrawn results from Ekblom *et al.* (124). Results from the transfusion of additional blood are shown in dark red circles and those after blood withdrawal in light red circles. Adapted with permission from Ekblom B, Goldbarg AN, Gullbring B. Response to exercise after blood loss and reinfusion. J Appl Physiol 1972;33:175–180.

Increasing the amount of hemoglobin in the blood has the biological effect of increasing oxygen transport from lungs to tissues, where the increased availability of oxygen enhances aerobic energy expenditure. This is exploited to its greatest effect in endurance sports (1). The experiments of Ekblom *et al.* (124) in 1972 (Fig. 4) demonstrated strong linear relationships between changes in hemoglobin [due to withdrawal or retransfusion of 1, 2 or 3 U (400 mL) of blood] and aerobic capacity, established by repeated testing of maximal exercise-induced oxygen consumption before and after each procedure (124). As already noted, circulating hemoglobin levels are on average 12% higher in men than women (125). It may be estimated that as a result the average maximal oxygen transfer will be ∼10% greater in men than in women, which has a direct impact on their respective athletic capacities.

#### Observational data

The proposition that the sex difference in circulating hemoglobin levels is likely to be due to the sex difference in average circulating testosterone concentrations is supported by the fact that male castration (*e.g.*, for advanced prostate cancer) (126) and androgen deficiency due to reproductive system disorders (127) reduce circulating hemoglobin in men, eliminating the sex difference, whereas testosterone replacement therapy restores circulating hemoglobin to adult male levels (121, 127, 128).

**Figure 5.**
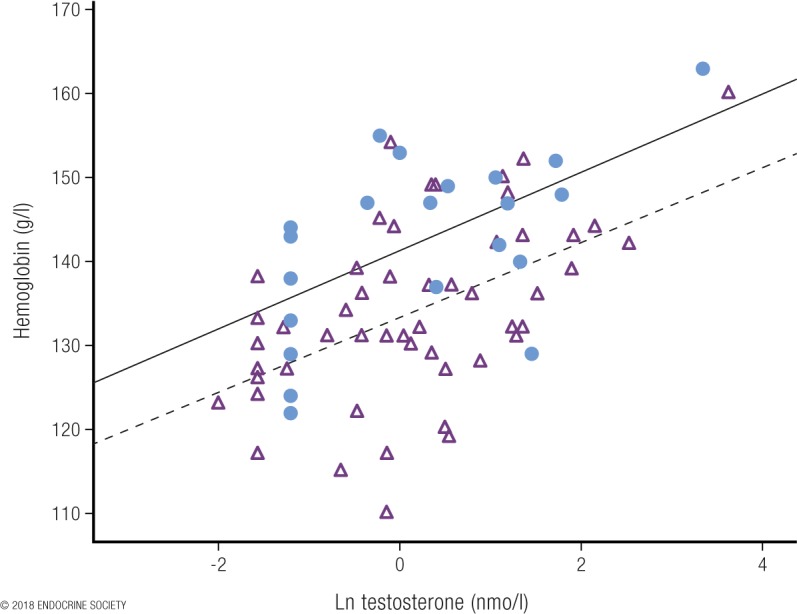
Plot of circulating hemoglobin against the natural logarithm of serum testosterone in women with congenital adrenal hyperplasia [from Karunasena *et al.* (92)]. The filled circles represent a cohort where serum testosterone was measured by immunoassay. The open triangles denote a second cohort, where serum testosterone was measured by LC-MS. Note the systematic overestimation of testosterone by the immunoassay used in cohort 1 vs LC-MS measurement in cohort 2. Despite that overestimation, however, the correlations were similar in both cohorts. Reproduced under a Creative Commons BY-NC-ND 4.0 license from Karunasena N, Han TS, Mallappa A, *et al.* Androgens correlate with increased erythropoiesis in women with congenital adrenal hyperplasia. Clin Endocrinol (Oxf) 2017;86:19–25.

An unusually informative observational study of women with CAH provides unique insight into testosterone effects on circulating hemoglobin in otherwise healthy women (92). Women with CAH require glucocorticoid replacement therapy but exhibit widely varying levels of hormonal control (79). The degree of poor control is associated with increasing levels of circulating testosterone ranging from normal female concentrations up to 36 nmol/L, and these levels correlate closely (*r* = 0.56) with levels of circulating hemoglobin (Fig. 5). Interpolating from the dose-response regression, increases in circulating testosterone measured by LC-MS from 0.9 nmol/L to 5 nmol/L, 7 nmol/L, 10 nmol/L, and 19 nmol/L were associated with increases in circulating hemoglobin of 6.5%, 7.8%, 8.9%, and 11%, respectively, establishing a strong dose-response relationship. An 11% increase in circulating hemoglobin translates to a 10% difference in maximal oxygen transfer (124), which may account for virtually all the 12% sex difference in male and female circulating hemoglobin (125). To put this into context, any drug that achieved such increases in hemoglobin would be prohibited in sports for blood doping, as this difference is sufficient to have ergogenic effects, even without taking into account any testosterone effects on muscle mass or strength (for which data were not available in that study). Conversely, among elite female athletes with circulating testosterone in the healthy premenopausal female range, circulating hemoglobin does not correlate with athletic performance (35). In women with the mild hyperandrogenism of PCOS, circulating hemoglobin and hematocrit are reported as not (129) or marginally increased (130), findings that may be influenced by the fact that PCOS is associated with reduced or absent menstruation, thereby reducing the iron loss of regular menstruation.

#### Interventional data

**Figure 6.**
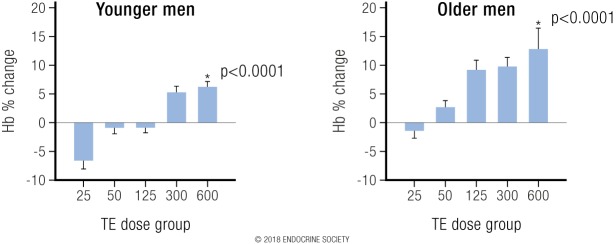
From Coviello *et al.* (131): Depicts the strong dose-response relationship between increasing testosterone dose with resulting change in blood hemoglobin in young and older men. Reproduced with permission from Coviello AD, Kaplan B, Lakshman KM, *et al.* Effects of graded doses of testosterone on erythropoiesis in healthy young and older men. J Clin Endocrinol Metab 2008;93:914–919.

In the Bhasin *et al*. (111) studies, in both young and older men the highest testosterone dose produced a 12% increase in blood hemoglobin compared with the lowest dose, reflecting a strong dose-response relationship (Fig. 6) (131). Analogous findings were reported for testosterone treatment effects in postmenopausal women where the highest dose (25 mg weekly) of testosterone, which increased mean serum testosterone to 7.3 nmol/L, had the largest increase (3%) in blood hemoglobin and hematocrit (112).

Corroborative findings are available from studies of transmen (F2M transgender), that is, natal females who subsequently receive testosterone treatment at replacement doses to create adult male circulating testosterone concentrations, who exhibit increases in circulating hemoglobin to male levels [reviewed in (132–134)]. Testosterone treatment in 17 (F2M) transmen that created mean circulating testosterone levels of 31 nmol/L also increased hemoglobin levels by 15% (114). Conversely, one prospective 12-month study of transgender (nonathlete) individuals reported that testosterone suppression (by an estrogen-based regimen) to normal female levels in 20 (M2F) transwomen reduced hemoglobin by 14%.

If such an increase in hemoglobin were produced by any chemical substance, it would be considered doping, according to the World Anti-Doping Code.

### Bone

#### Biology

There is extensive experimental evidence from genetic mouse models showing that the sex differences in bone size, mass, and function are due to the sex difference in circulating testosterone. These effects have been reported from studies of global and tissue or cell-selective inactivation of ARs or estrogen receptors that show that androgen effects are mediated by both direct effects on the AR as well as indirect effects mediated via aromatization of testosterone to estradiol to act on estrogen receptors [reviewed in (135)]. Bone grows in length due to epiphyseal chondral growth plates that provide cartilage, forming the matrix for lengthening of long bone, which is terminated by an estrogen-dependent mechanism that depends on aromatization of testosterone to estradiol. Similarly, bone width and density are increased through appositional growth from periosteal and endosteal expansion that depend on bone loading and androgen exposure together with other factors. An important difference between androgen effects on bone compared with effects on muscle or hemoglobin is that developmental bone effects of androgens are likely to be irreversible.

#### Observational data

Men have distinctively greater bone size, strength, and density than do women of the same age. As with muscle, sex differences in bone are absent prior to puberty but then accrue progressively from the onset of male puberty due to the sex difference in exposure to adult male circulating testosterone concentrations [reviewed in (135)]. The earlier onset of puberty and the related growth spurt in girls as well as earlier estrogen-dependent epiphyseal fusion explains shorter stature of girls than boys. As a result, on average men are 7% to 8% taller with longer, denser, and stronger bones, whereas women have shorter humerus and femur cross-sectional areas being 65% to 75% and 85%, respectively, those of men (106). These changes create an advantage of greater bone strength and stronger fulcrum power from longer bones. Additionally, whereas passing through puberty enhances male physical performance, the widening of the female pelvis during puberty, balancing the evolutionary demands of obstetrics and locomotion (136, 137), retards the improvement in female physical performance, possibly driven by ovarian hormones rather than the absence of testosterone (138, 139).

Sex differences in height have been the most thoroughly investigated measure of bone size, as adult height is a stable, easily quantified measure in large population samples. Extensive twin studies show that adult height is highly heritable with predominantly additive genetic effects (140) that diverge in a sex-specific manner from the age of puberty onwards (141, 142), the effects of which are likely to be due to sex differences in adult circulating testosterone concentrations.

Bone density (total and medullary cross-sectional area) is increased in women with CAH with variably elevated serum testosterone (including into the male range) when it is only partially suppressed by glucocorticoid treatment (143), although more effective glucocorticoid suppression lowers bone density (144).

#### Interventional data

Well-designed, placebo-controlled direct interventional studies of supraphysiological androgen effects on bone in females are few, rarely feasible, and unlikely to be performed for ethical and practical reasons. Unlike muscle, which responds relatively rapidly to androgen effects so that muscle studies in humans can be completed within 3 to 4 months (65, 111, 112, 119, 145), comparable bone studies would typically take a year or more to reach plateau effects. Hence, such direct investigational studies in otherwise healthy women would risk side effects of virilization that may be only slowly and partly reversible, if at all, as well as potential promotion of hormone-dependent cancers making such studies ethically and practically not feasible.

#### Effects on athletic performance

The major effects of men’s larger and stronger bones would be manifest via their taller stature as well as the larger fulcrum with greater leverage for muscular limb power exerted in jumping, throwing, or other explosive power activities. The greater cortical bone density and thereby resistance to long bone fractures is unlikely to be relevant to the athletic performance of young athletes, in whom fractures during competition are extremely rare and not expected to be linked to sex. Alternatively, stress fractures in athletes, mostly involving the legs, are more frequent in females with the male protection attributable to their larger and thicker bones (146).

### Other androgen-sensitive sex dichotomous effects

#### Biology and observational data

Many if not most other aspects of physiology exhibit sex differences and may therefore enhance the impact of the male advantage in sports performance of the dominant determinants (muscle and hemoglobin). Examples include sex differences in exercise-induced cardiac (147, 148) and lung (149) function and mitochondrial biogenesis and energetics (95). However, the limited knowledge of the magnitude and hormonal mechanisms involved, specifically the degree of androgen dependence of these mechanisms, means that it is difficult to estimate their contribution, if any, toward the sex difference in athletic performance. The sex difference in pulmonary function may be largely explained by the androgen-sensitive sex difference in height, which is a strong predictor of lung capacity and function (149). Further physiological studies of the androgen dependence of other physiological sex differences are awaited with interest.

Psychological differences between men and women on mental function (*e.g.*, rotational orientation) (150) as well as mood, motivation, and behavioral effects may involve androgen-sensitive effects during prenatal and perinatal as well as postpubertal effects (151, 152).

#### Interventional data

There is some limited direct evidence from well-designed, placebo-controlled trials that administration of testosterone or other androgens at supraphysiological doses directly affect mood and behavior, notably inducing hypomania (153). In a randomized placebo-controlled study of testosterone administration in postmenopausal women (112), in case of those receiving the highest dose (the only one causing circulating testosterone levels to exceed the normal female range), there was not only an increase in muscle mass (4.4%) but a strikingly greater increase in muscle strength (12% to 26%), suggesting an enhanced mental motivational effect of testosterone on the effort-dependent tests of muscle strength.

## Alternative Mechanisms Proposed to Explain Sex Differences in Athletic Performance

Alternative explanations for the sex difference in athletic performance, other than it being due to the sex difference in postpubertal circulating testosterone, have been proposed, including (1) sex differences in height because height is a predictor of muscle mass (116), (2) genetic sex differences due to the influence of unspecified Y chromosome genes (154), and (3) sex differences in GH secretion (116).

### Effects of height

One proposal has been that, as men are taller than women, height differences may explain the sex differences in muscle mass and function, which explains some athletic success (116). Numerous factors contribute to the regulation of adult muscle mass, including genetics, race, adiposity, hormones, physical activity (exercise/training), diet, birth order, and bone size (including height) [reviewed in (155)]. Among the nonhormonal factors, genetics explains a large proportion [∼50% to 60% from pooled twin studies (156)] of the variability in muscle mass and strength (157, 158) and may be explained in turn by the equally high genetic contributions to circulating testosterone (37, 38). Some factors influencing muscle mass and strength such as physical activity, adiposity, and bone size are also partly androgen-dependent. Prior to puberty there is no sex difference in skeletal features, including height (159, 160). However, with the onset of puberty, girls aged 11 and 12 years are transiently taller than peer-aged boys due to their earlier onset of the female pubertal growth spurt, but from the age of 14 years onward the taller stature in males emerges and stabilizes (141). Hence, similar to muscle mass, sex differences in bone size (including length, density, and height) arise after male puberty establishes the marked dichotomy between men and women in adult circulating testosterone concentrations. Taller height is advantageous in some sports (basketball, some football codes, combat sports), but in others (horse racing jockeys, cycling, gymnastics, weightlifting, bodybuilding) short stature provides a greater power/strength-to-weight ratio as well as superior rotational balance, speed, and agility. However, the male advantages in speed, strength, and endurance apply regardless of whether height is advantageous. Hence, the sex differences in height, where they exist, are largely dependent on postpubertal differences in circulating testosterone when sex differences in height are first expressed.

### Genetic effects of Y chromosome

It has also been proposed that the sex difference in athletic performance may be due to genetic effects of an unspecified Y chromosome gene that may dictate taller stature (154), as height is correlated with men’s greater muscle mass. The small human Y chromosome has few functional genes and none with a known effect on height other than the short stature homeobox (SHOX) gene, located in the pseudoautosomal regions of the tip of the short arms of X and Y chromosomes (161). Adult height displays an apparent dose dependency on SHOX gene copy number that is a major factor contributing to explaining both the short stature of 45,XO females (Turner syndrome), who have a single copy of the SHOX gene, as well as the tall stature of 47,XXY males (Klinefelter syndrome), who have three copies (161). However, when SHOX copy number is the same, men with additional supernumerary Y chromosomes (*e.g.*, 47,XYY) are the same height as 47,XXY men (162). Hence, there is no evidence supporting dosage-dependent Y chromosomal gene effects on height independent of SHOX gene copy number, nor does men’s possession of a Y chromosome explain the height difference between adult men and women. On the contrary, the tall stature of 47,XXY men is at least partly due to the concomitant androgen deficiency leading to pubertal delay. Pubertal delay prolongs long bone growth due to delayed epiphyseal closure, an estrogen-dependent effect that requires adequate production of testosterone as a substrate for aromatization to estradiol, resulting in tall stature. Similar eunuchoidal features and taller stature are evident in 46,XY men with congenital hypogonadotropic hypogonadism (Kallmann syndrome and its variants) with comparable congenital onset of androgen deficiency, also manifest as pubertal delay and long bone overgrowth. Hence, taller height is better explained by impaired testicular function with delayed puberty and epiphyseal closure rather than unspecified Y chromosome dosage effects. In any case, rare aneuploidies in themselves do not explain the sex difference in height in the general population of individuals with normal sex chromosomes.

### Growth hormone

The proposal that the sex difference in muscle mass and function might be due to sex differences in endogenous GH secretion (116) is refuted by the extensive and conclusive clinical evidence that endogenous GH secretion in young women is consistently higher (typically twice as high) as in young men of similar age (163–170). Those findings cannot explain the male advantage in muscle mass and strength unless GH retards muscle growth/function, for which there is no evidence. Furthermore, estrogens inhibit GH-dependent, hepatic IGF-1 production, the major pathway of GH action (171, 172). The weak observational association between low circulating IGF-1 and some, but not other, measures of weak muscle strength and limited mobility among older women may reflect general age-associated debility rather than any specific hormonal effects (173). Finally, the evidence that endogenous GH plays no role in sex differences in muscle mass and function is supported by evidence from the most extensive interventional study of GH treatment to non-GH–deficient adults, daily GH administration for 8 weeks to healthy recreational athletes produced only marginally significant improvement in exercise performance of men and none in women (174). These findings are consistent with the speculation that GH (or IGF-1) may be an amplifier of testosterone effects and therefore be a consequence of the sex difference in circulating testosterone rather than its cause.

## The Impact of Adult Male Circulating Testosterone Concentrations on Sports Performance

Plausible estimates of the magnitude of the ergogenic advantage of adult male circulating testosterone concentrations are feasible from the limited available observational and interventional studies.

Population data on the ontogeny of puberty show that prior to puberty boys and girls have comparable athletic performance, whereas sex differences in athletic performance emerge coinciding with the rise in circulating testosterone from the onset of male puberty. Male puberty results in circulating testosterone concentrations rising from the prepubertal and female postpubertal range (<2 nmol/L) to adult male circulating testosterone concentrations (18). This is associated with a 10% to 12% better performance in running and swimming events and 20% enhancement in jumping events (8).

A minimal estimate of the impact of adult male testosterone concentrations on muscle size and strength in females is provided by the Huang *et al.* (112) study of postmenopausal women. In this study the highest testosterone dose (weekly injections of 25 mg of testosterone enanthate) increased mean circulating testosterone from 0.9 nmol/L to 7.3 nmol/L, which is equivalent to the circulating testosterone of boys in early to middle puberty. After 24 weeks of testosterone treatment, the increase in circulating testosterone concentrations led to significant increases in muscle size of 4.4% and in muscle strength of 12% to 26%. Given the limited testosterone dose (and concentration) as well as study duration, it is likely that these findings underestimate the magnitude of the impact that sex difference in circulating testosterone has on muscle mass and strength, and therefore on athletic performance.

**Figure 7.**
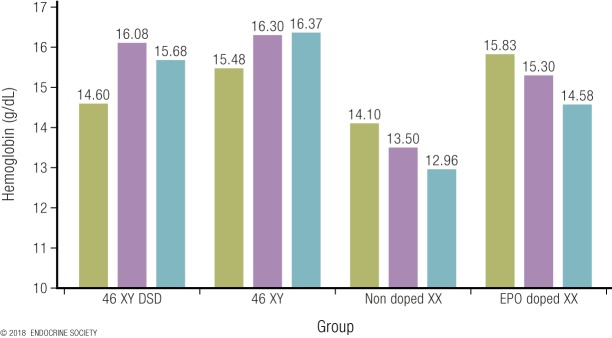
Mean hemoglobin concentrations (g/dL) of 12 elite athletes in 4 groups of 3 XY or XX middle-distance runners. The hemoglobin concentrations were collected as a part of the Athlete Biological Passport and analyzed according to the World Anti-Doping Agency standard methods. Each bar (athlete) is the mean of a minimum of three blood samples. In the 46,XY DSD group, blood was collected in a period when the athlete was not undergoing hormonal suppressive treatment.

Converse effects of reduced athletic performance in athletes who undergo suppression of circulating testosterone concentrations from those in the male into the female range have been reported. Among recreational (nonelite) athletes, an observational study showed a consistent deterioration in athletic performance of transwomen (M2F transgender) athletes corresponding closely to the suppression of circulating testosterone concentrations (175). Similarly, among elite athletes with circulating testosterone in the male range due to DSDs, comparable findings of athletic performance reduced by an average of 5.7% when circulating testosterone was suppressed from the male range to <10 nmol/L (176). Subsequently, when the IAAF hyperandrogenism rule was suspended in 2015, and so these elite athletes could train and compete with unsuppressed serum testosterone levels, their athletic performances increased by a similar amount. Additionally, circulating hemoglobin levels in these untreated DSD athletes were comparable with male athletes or with female athletes doping with erythropoietin (Fig. 7). However, when circulating testosterone was suppressed to <10 nmol/L the levels of circulating hemoglobin were 12% lower and again comparable with nondoped, non-DSD females, corresponding to the 12% magnitude of the sex difference in hemoglobin between men and women (125).

**Figure 8.**
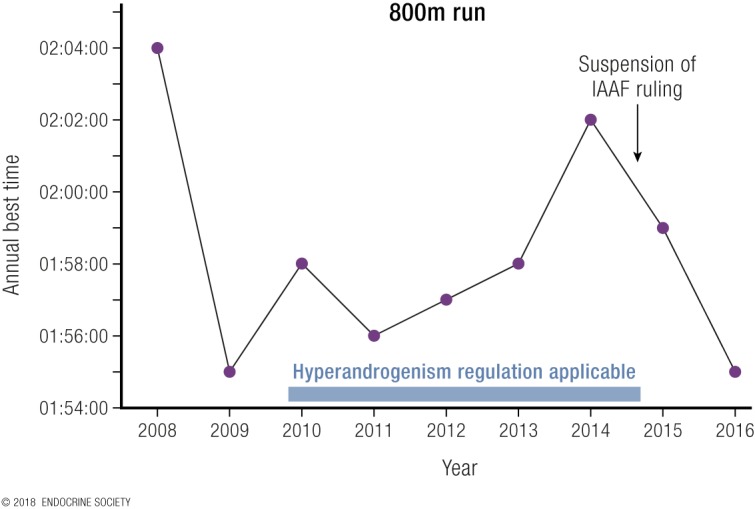
Best annual 800-m times of an elite female athlete between 2008 and 2016. Data provided by Dr. Richard Auchus, University of Michigan, Ann Arbor, Michigan.

Congruent findings are also known for an elite female athlete whose serial athletic performance based on publicly available best annual times between 2008 and 2016 for the 800-m running event are depicted in relationship to the original 2011 IAAF hyperandrogenism regulation (Fig. 8).

Based on the established dose-response relationships, suppression of circulating testosterone to <10 nmol/L would not eliminate all ergogenic benefits of testosterone for athletes competing in female events. For example, according to the Huang *et al.* (112) study, reducing circulating testosterone to a mean of 7.3 nmol/L would still deliver a 4.4% increase in muscle size and a 12% to 26% increase in muscle strength compared with circulating testosterone at the normal female mean value of 0.9 nmol/L. Similarly, according to the Karunasena *et al.* (92) study, reducing circulating testosterone concentration to 7 nmol/L would still deliver 7.8% more circulating hemoglobin than the normal female mean value. Hence, the magnitude of the athletic performance advantage in DSD athletes, which depends on the magnitude of elevated circulating testosterone concentrations, is considerably greater than the 5% to 9% difference observed in reducing levels to <10 nmol/L.

The physiological mechanism underlying these observations is further strengthened by prospective controlled studies of initiation of cross-sex hormone treatment in transgender individuals (114, 177). These show that during the first 12 months muscle mass (area) was decreased by 9.4% and hemoglobin levels by 14% in 20 transwomen (M2F transgender) treated with an estrogen-based regimen that reduced circulating testosterone concentrations from the male range to the female range. Conversely, in 17 transmen (F2M transgender) treated for the first time with testosterone for 12 months (which increased circulating testosterone levels to a mean of 31 nmol/L), muscle mass increased by 19.2% and hemoglobin by 15% (114). The muscle mass findings remained stable between 1 and 3 years after initiation of treatment, although fat mass continued to change between 1 and 3 years of testosterone treatment (177). These studies did not report muscle strength, but other studies of testosterone dose-response relationships for muscle mass and strength show consistently positively correlation (65, 93, 117, 119), although with disproportionately greater effect on muscle strength than on muscle mass. Hence, the muscle mass estimates in these prospective treatment initiation studies in transgender individuals likely underestimate the muscle strength gains from elevated testosterone levels where the circulating testosterone markedly exceeds female range to be within the male range as occurs in severe hyperandrogenism of DSD females, poorly controlled transwomen (M2F transgender), or transmen (F2M transgender). These effects are also the biological basis of the ergogenic efficacy of androgen doping in women.

Finally, to put these competitive advantages into context, the winning margin (the difference in performance by which a competitor misses a gold medal, any medal, or making the final) in elite athletic or swimming events during the last three Olympics is <1% equally for both male and female events (Table 5).

**Table 5.** The Winning Margin in Elite Athletic or Swimming Events During the Last Three Olympics

Median Margin (%)*^a^*	n	Win Gold	Win Medal	Make Final
Athletics*^b^*				
Running	81	0.62	0.31	0.22
Jumping	24	0.92	0.42	0.92
Throwing	24	1.93	0.70	0.75
Swimming*^c^*				
Backstroke	12	0.56	0.28	0.16
Breaststroke	12	0.84	0.14	0.17
Butterfly	12	0.52	0.48	0.12
Freestyle	30	0.49	0.23	0.14
Relay	18	0.37	0.35	0.12

^a^ Winning margin is defined as the difference (expressed as a percentage of the faster time) between first and second place (Win Gold), between third and fourth place (Win Medal), and between the last into the final and the first that missed out (Make Final). Years (2008, 2012, 2016) and sexes were combined as there were no significant differences in winning margin between them.

^b^ Running includes 100 m, 200 m, 400 m, 800 m, 1500 m, 5000 m, 10,000 m, marathon, and 3000-m steeplechase, 110-m (male)/100-m (female) and 400-m hurdles, 4 × 100-m and 4 × 400-m relays, and 20-km and 50-km walk events. Jumping includes high jump, long jump, triple jump, and pole vault events. Throwing includes javelin, shot put, discus, and hammer events. Heptathlon and decathlon were not included as their final results are in points, not times.

^c^ Events comprise 100 m and 200 m for the form strokes and 50 m, 100 m, 200 m, 400 m, 800 m (female)/1500 m (male) and marathon 10 km, with the relays being the 4 × 100-m medley and 4 × 100-m and 4 × 200-m freestyle relays.

## Gaps in Knowledge and Research Limitations

The major limitations on scientific knowledge of the impact of adult male circulating testosterone concentrations on the sex difference in athletic performance is the lack of well-designed studies. Ideally, these would need to replicate adult male circulating testosterone concentrations for sufficient time in women to investigate the effects on muscle, hemoglobin, bone, and other androgen-sensitive measures that display consistent sex dichotomy in the population. However, the ethical and safety concerns preventing such studies hitherto are likely to remain formidable obstacles due to the risk of unacceptable and potentially irreversible virilization as well as of promoting hormone-dependent cancers in women.

With the exception of one interventional study administering a relatively low testosterone dose (*i.e.*, low for males) to women (112), the available evidence comprises observational studies that can only examine the effects of serum testosterone within physiological female limits or sparse and mostly uncontrolled data from intersex/DSD athletes. Although the available observational findings in healthy females are informative, the key question is the magnitude and dose response of effects at still higher circulating testosterone concentrations on the performances of women. Whereas a testosterone dose-response relationship has been established in women at relatively low (for men) testosterone dose and circulating concentrations, it remains unproven (even if clearly plausible) that the testosterone dose-response relationships established in men for muscle, hemoglobin, and bone can be extrapolated to women when they are exposed to higher circulating testosterone concentrations (*i.e.*, comparable with male levels). It is theoretically possible there could be differences between men and women in muscle responses to testosterone, as muscle cell populations might express genetic differences in androgen sensitivity (for which there are no data), or alternatively the long-term prior pattern of testosterone exposure from conception to adulthood might lead to differences in testosterone dose responsiveness after maturity. Although the dose-response relationship in women may be similar to what is seen in men, there is also anecdotal evidence that the dose-response curves may be left shifted so that testosterone has greater potency in women than in men at comparable doses and circulating levels. The prediction is supported by the anecdotal evidence from the surreptitious East German national doping program in which the supervising doctors asserted from their experience of illicit cheating that androgens had more potent ergogenic effects in women than in men (120), a speculative opinion shared by many experienced sports medicine physicians.

There is no known means of increasing endogenous testosterone in women to anything like the requisite degree to attempt to answer these questions. In healthy men, circulating testosterone originates almost exclusively from a single source (testicular Leydig cells) and is subject to tight hypothalamic negative feedback control, so that either direct stimulation (by human chorionic gonadotropin) or indirect reflex effects (*e.g.*, from estrogen blockers operating via negative feedback) to enhance Leydig cell testosterone secretion are feasible. However, similar mechanisms do not operate in women, in whom circulating testosterone originates from three different sources (adrenal, ovary, extraglandular conversion of androgen precursors), none of which is subject to tight testosterone negative feedback control. As a result, it is not feasible to produce a sufficient increase in circulating testosterone in women either by direct ovarian stimulation or indirect reflex effects to test this hypothesis even if doing so were deemed ethical and safe. Alternatively, carefully controlled, graded-dose studies in F2M transgender individuals might be informative but are largely lacking at this time.

Hence, the only feasible design of such studies would be testosterone (or another androgen) administration to healthy young women. The only well-designed, placebo-controlled study of testosterone in otherwise healthy postmenopausal women was restricted to relatively low testosterone doses that, although clearly supraphysiological for women, were only 20% to 25% of male testosterone replacement doses (112). We are currently performing a double-blind, randomized, placebo-controlled study of the effects of moderately increased testosterone concentration on physical performance and behavior in young healthy women (ClinicalTrials.gov no. NCT03210558). However, obtaining ethical approval to administer supraphysiological testosterone doses that maintain circulating testosterone in the male range for sufficiently prolonged periods, as well as the practical difficulties in recruitment, are likely to remain obstacles to definitive resolution of this question.

In men, analogous ethical concerns over short- and long-term adverse effects delayed the definitive studies of supraphysiological testosterone doses to healthy young and older men but were eventually overcome. This was despite the fact that, uniquely among hormones, there is no known disease state in men due to pathologically excessive testosterone secretion. In contrast, in women, supraphysiological testosterone effects are known to produce virilization side effects that may be only slowly and partially, if at all, reversible. However, maintaining clearly supraphysiological testosterone concentrations would require treatment of months (muscle) or years (bone) and would replicate not only a known hyperandrogenic disease state (PCOS) but also potentially increasing risk of hormone-dependent cancers. In these circumstances, it could only be justifiable to replicate in women the salient testosterone dose-response studies available from men if the available evidence of dose-response relationship in men was not sufficiently convincing and/or there was reason to think that these dose-response characteristics would be substantially different in women. Overall, the unequivocal dose-response evidence in men together with the available overlap evidence in women appears sufficiently persuasive, so that it is doubtful that women would respond differently from men if their circulating testosterone levels were raised to the male range. More broadly, there is no more reason to require separate studies in women vs men than there is for every different ethnic subgroup of people. An aesthetic preference for splitting categories is not a sound reason to require the virtually impossible standard of establishing fresh and comprehensive empirical evidence in women of testosterone dose-response effects ranging into male circulating testosterone concentrations.

An analogy can be drawn to the World Anti-Doping Agency’s practice of accepting salient surrogate evidence for banning the plethora of existing and new drugs with potential but individually unproven ergogenic effects where it is not feasible or ethical to require direct proof of the ergogenic effects. In that context, the firmly established ergogenic efficacy of androgens (on muscle mass and strength) and increased hemoglobin (on endurance) [evidence reviewed in (1)] mean that chemical substances or methods that increase endogenous testosterone, erythropoietin, or hemoglobin are also considered ergogenic (178). By parity of reasoning, if a condition causes a female athlete’s circulating testosterone levels to be in the male range, well exceeding normal female levels, with consequential increases in muscle, hemoglobin, and bone effects (at least), an ergogenic effect may reasonably be assumed.

## Conclusions

The available, albeit incomplete, evidence makes it highly likely that the sex difference in circulating testosterone of adults explains most, if not all, the sex differences in sporting performance. This is based on the dose-response effects of circulating testosterone to increase muscle mass and strength, bone size and strength (density), and circulating hemoglobin, each of which alone increases athletic capacity, as well as other possible sex dichotomous, androgen-sensitive contributors such as mental effects (mood, motivation, aggression) and muscle myoglobin content. These facts explain the clear sex difference in athletic performance in most sports, on which basis it is commonly accepted that competition has to be divided into male and female categories.

The first IAAF hyperandrogenism regulation specified a hormonal eligibility criterion of a serum testosterone of <10 nmol/L for an androgen-sensitive athlete’s participation in the protected category of female athletic events. This threshold was based on serum testosterone measurements by immunoassays. However, no reliable method-independent consensus threshold could be established using commercial testosterone immunoassays, as these assays differ systematically due to method-specific bias arising unavoidably from the specificity of the different proprietary antibodies employed (25). Based on measurements using the more accurate and specific mass spectrometry methods, if the objective is to require female athletes with congenital conditions that cause them to have serum testosterone concentrations in the normal male range to bring those levels down to the same range as other female athletes, then (allowing for PCOS athletes) the threshold used should not be >5.0 nmol/L. This represents a conservative criterion that includes all healthy young (<40 years) women, including those with PCOS. Conversely, this criterion is generous to intersex/DSD females in allowing them to maintain a higher serum testosterone (2 to 5 nmol/L) than most non-PCOS competitors in female events even though increases in muscle mass and strength and hemoglobin would be expected in this range. This is so even though the range remains below the circulating testosterone levels of middle male puberty when the major biological effects of men’s higher circulating testosterone begin to be fully expressed. Ongoing compliance with the eligibility criterion is also an important variable because the estrogen-based suppression of circulating testosterone, typically using daily administered estrogen products, has a rapid onset and offset. Adequate monitoring to prevent gaming of eligibility criteria would require regular random rather than announced blood sampling.

A related matter is how long such a threshold of circulating testosterone should be maintained prior to competition. In both intersex/DSD and transgender individuals, the developmental effects of adult male circulating testosterone concentrations will have established the sex difference in muscle, hemoglobin, and bone, some of which is fixed and irreversible (bone size) and some of which is maintained by the male circulating testosterone concentrations (muscle, hemoglobin). The limited available prospective evidence from initiation of transgender cross-sex hormone treatment suggests that the advantageous increases in muscle and hemoglobin due to male circulating testosterone concentrations are induced or reversed during the first 12 months and the androgenic effects may plateau after time. This time course is much faster than the somatic effects of male puberty, which evolve over years and for some variables (*e.g.*, peak bone mass) are not complete for up to a decade after the start of puberty. However, the abrupt hormonal changes induced by medical treatment in intersex/DSD or transgender individuals may be telescoped compared with male puberty where circulating testosterone concentrations increase irregularly and incompletely for some years. Additional data are available from the unique investigative model of men undergoing castration for prostate cancer. Just as androgen sensitivity to testosterone may differ between tissues (65), the time course of offset of androgen effects following withdrawal of male testosterone concentrations may also differ between the major androgen-responsive tissues. For example, circulating hemoglobin shows a progressive fall for 6 months reaching a nadir and plateau at 12 to 16 months in six studies involving 534 men undergoing medical castration for prostate cancer (179–184). Although these studies of older men with prostate cancer must be extrapolated with caution, age, stage of disease, race, and baseline circulating testosterone concentration did not affect the rate or extent of decline in hemoglobin (179, 181). Comparable longitudinal studies of muscle loss, strength, and performance following castration for prostate cancer are well summarized (185), showing progressive loss for 24 months (see Fig. 4). Further clinical studies to define the time course of changes, mainly offset, in testosterone-dependent effects, notably on muscle and hemoglobin, are badly needed to determine the optimal duration for cross-sex hormone effects in sports.

## Abbreviations

AR: androgen receptor
CAH: congenital adrenal hyperplasia
CAIS: complete androgen insensitivity syndrome
DSD: disorder (or difference) of sex development
F2M: female-to-male
IAAF: International Association of Athletic Federations
IOC: International Olympic Committee
LC-MS: liquid chromatography–mass spectrometry
M2F: male-to-female
PAIS: partial androgen insensitivity syndrome
PCOS: polycystic ovary syndrome
SHOX: short stature homeobox
